# Robotic TAPP inguinal hernia repair: lessons learned from 97 cases

**DOI:** 10.1590/0100-6991e-20202704

**Published:** 2021-01-26

**Authors:** ANDRE LUIZ GIOIA MORRELL, ALEXANDER CHARLES MORRELL, JOSE MAURICIO FREITAS MENDES, ALLAN GIOIA MORRELL, ALEXANDER MORRELL

**Affiliations:** 1 - Instituto Morrell, Cirurgia Robótica e Minimamente Invasiva do Aparelho Digestivo - São Paulo - SP - Brasil; 2 - Sociedade Beneficente Israelita Brasileira Hospital Albert Einstein, Cirurgia Geral e Aparelho Digestivo Minimamente Invasiva e Robótica - São Paulo - SP - Brasil; 3 - Rede D’Or Hospital São Luiz, Cirurgia Robótica e Minimamente Invasiva do Aparelho Digestivo - São Paulo - SP - Brasil; 4 - Vila Nova Star - Rede D’Or Hospital São Luiz, Cirurgia Robótica e Minimamente Invasiva do Aparelho Digestivo - São Paulo - SP - Brasil; 5 - Hospital Alemão Oswaldo Cruz, Cirurgia Geral e do Aparelho Digestivo Minimamente Invasiva e Robótica - São Paulo - SP - Brasil; 6 - Grupo Leforte, Cirurgia Robótica e Minimamente Invasiva do Aparelho Digestivo - São Paulo - SP - Brasil

**Keywords:** General Surgery, Hernia, Hernia, Inguinal, Robotics, Robotic Surgical Procedures, Minimally Invasive Surgical Procedures, Cirurgia Geral, Hérnia Inguinal, Hérnia, Robótica, Procedimentos Cirúrgicos Robóticos, Procedimentos Cirúrgicos Minimamente Invasivos

## Abstract

**Objectives:**

minimally invasive inguinal hernia repair has proven advantages over open procedures including less pain and earlier return to normal activity. Robotic surgery adds ergonomics, a three-dimensional high definition camera and articulating instruments overcoming some laparoscopic limitations. We aimed to report the outcomes of the early experience of over 97 robotic inguinal hernia repairs performed by a referred surgical group in Brazil.

**Methods:**

a review of a prospective mantined database was conducted in patients submitted to robotic transabdominal preperitoneal (TAPP) inguinal hernia repairs between March 2016 and February 2020. Descriptive statistics were performed. Surgical outcomes data and patient follow-ups are reported.

**Results:**

retrospective chart review identified 97 patients submitted to robotic TAPP inguinal hernia repair. Mean age was 36.4 years, with median BMI of 26.9 kg/m^2^. Mean console time was 58 min (range 40-150) and patients were discharged within 24 hours of their stay in a majority of cases. Mesh was placed in all procedures and there were no conversion rates. Complications were low grade and no recurrence was seen after a mean follow-up of 642 days.

**Conclusion:**

this study represents to-date the first brazilian case series of robotic TAPP inguinal hernia repair. Our results encourage that robotic assisted TAPP inguinal hernia repair appears to be technically feasible and safe in experienced hands, with good outcomes achieving high health-related quality of life and low recurrence rates in the short and long term.

## RESUMO

**Objetivo:**

a cirurgia minimamente invasiva das hérnias inguinais apresenta vantagens comprovadas em relação aos procedimentos convencionais, incluindo menos dor e retorno precoce às atividades rotineiras. A cirurgia robótica acrescenta uma melhor ergonomia, ótica estável tridimensional de alta definição e instrumentos articulados, superando algumas limitações laparoscópicas. O objetivo deste estudo é relatar os resultados da experiência inicial de 97 cirurgias de correção de hérnias inguinais robóticas pela técnica transabdominal pré-peritoneal (TAPP) realizadas por um grupo cirúrgico referência no Brasil.

**Métodos:**

foi realizada uma revisão de um banco de dados mantido prospectivamente de pacientes submetidos a cirurgias de hérnia inguinal TAPP robótica entre março de 2016 e fevereiro de 2020. Uma análise detalhada dos resultados foi feita e dados dos resultados cirúrgicos e acompanhamento dos pacientes são relatados.

**Resultados:**

a revisão retrospectiva do prontuário identificou 97 pacientes submetidos à cirurgia de hérnia inguinal TAPP robótica. A idade média foi de 36,4 anos, com IMC médio de 26,9 kg/m^2^. O tempo médio de procedimento cirúrgico no console robótico foi de 58 minutos. A colocação de prótese foi realizada em todos os procedimentos e não houve taxas de conversão ou intercorrências.

**Conclusão:**

este estudo representa a primeira série de casos de cirurgia robótica de hérnia inguinal pela técnica transabdominal pré-peritoneal no Brasil. Os resultados descritos reforçam que a hernioplastia inguinal robótica apresenta-se como tecnicamente viável e segura em mãos experientes, com bons resultados quanto a qualidade de vida e baixas taxas de recidiva a curto e longo prazo.

## INTRODUCTION

Inguinal hernia repair is still one of the most common surgical procedures performed worldwide. During the early 1990s the laparoscopic method of mesh implantation in the preperitoneal space was introduced^1^. The benefits of the minimally invasive surgery (MIS) became obvious, offering reduced wound complications, shorter hospital stays, improved pain control and expedited functional recovery^2^. Also, endoscopic repair also offers clear advantages in bilateral inguinal hernias and recurrent defects, allowing a wider and more reliable view of the posterior inguinofemoral anatomy. Transabdominal preperitoneal (TAPP) and totally extraperitoneal (TEP) approaches have gained space so the preperitoneal space started to be endorsed in minimally invasive hernia repair^3^. Despite these clearly documented advantages and published guidelines, the laparoscopic inguinal hernia repair has not been popularized among the surgeons and the growth of the technique remained flat for years^4^. Thinking about why so many surgeons have failed to adopt laparoscopic inguinal hernia repair as their procedure of choice, could probably be related to the requirement of advanced laparoscopic technique and a long learning curve, different from others minimally invasive procedures such as cholecystectomies^5^. Also, learning the anatomy of the posterior approach and the details required to safely complete repair is a paradigm shift in hernia procedures for general surgeons. 

Robotic surgery has gained popularity with potential dexterity, safety and cosmetic benefits. Some of its benefits have already been documented in urology and colorectal surgery^6^. Robotic surgery provides solutions to the challenges posed by laparoscopy, including wristed instruments, ease of intracorporeal suturing, and ergonomic advantages. Regarding hernia repair, anterior and inguinofemoral abdominal wall dissection difficulties are dramatically overcomed with the robotic-assisted procedures, enhancing surgeons ergonomics as well as higher image definition and freedom of movements. Escobar Dominguez et al.^7^ described the first robotic inguinal hernia repair showing its feasibility. Worldwide, the robotic platform has made the procedure more reproducible and allowed a safer and faster growth in MIS inguinal hernia repair. However, in the Brazilian scenario, the introduction of robotic technology into hospitals still encounters cost issues regarding all surgical areas. Training robotic surgeons is a highly demanding and expensive task, requiring the robotic platform, instrumentals and surgical proctors, not easily available all over the country. The purpose of this study is to report the first brazilian case-series of patients submitted to robotic TAPP inguinal hernia repair and its early outcomes by a referred surgical group.

## METHODS

This is a retrospective review of a prospective maintained database of all robotic TAPP inguinal hernia repairs performed by a single surgical group between March 2016, when the first case was performed, and February 2020. No laparoscopic or totally extraperitoneal access for hernia repairs were included in this study. Data was collected including patient demographics, preoperative risk factors, hernia characteristics (type, localization, recurrent) intraoperative variables (console time, mesh dimensions and area, mesh fixation) and postoperative outcomes (length of stay, wound-related and non-wound-related complications, 30-day readmissions, neuralgia, ischemic orchitis, recurrence) as well as the follow-up period. Minimally invasive hernia surgery benefits regarding bilateral defects and previous anterior repair is consensus. We do recognize that these are some relative contraindications such as previous laparoscopic repair or preperitoneal urologic procedure. However, with the advent of robotics in our current hernia repairs, we have become more liberal in our patient selection and comfortable even in more complex cases to opt for the robotic approach.

### Patient preparation and ports placement

**Figure 1:**
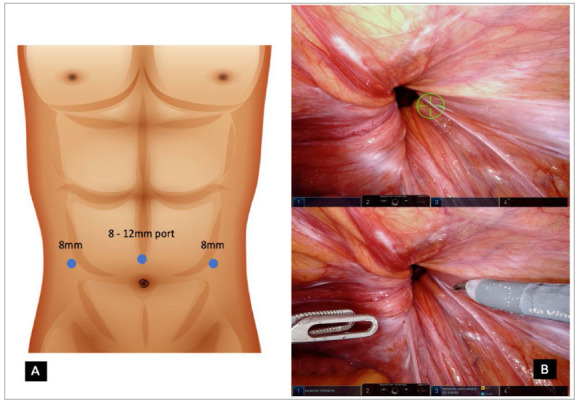
1A - Abdominal port placement. 1B - Robotic view of Inguinal defect and targeting area.

Under general anesthesia, patients are positioned supine with arms close to the trunk. Antibiotic prophylaxis is routinely used with administration of 1 g intravenous cefazolin during anesthetic induction. Foley catheter insertion isn’t mandatory. A small supraumbilical incision is made and pneumoperitoneum is achieved by a Veress needle puncture and carbon dioxide insufflation. A camera port is inserted into the abdominal cavity and two 8 mm robotic ports are placed either side lateral to the umbilicus, slightly above or below the umbilical imaginary line, with a minimum of 10 cm lateral to the supraumbilical port and 10 cm superior to the anterior superior iliac spine (ASIS) (Figure 1A). Whenever utilizing the da Vinci Si platform (Intuitive Surgical Inc. Sunnyvale, CA, USA), a 12mm camera port is used and a 30 degrees camera is inserted looking up followed by instrument placement. In the da Vinci Xi platform, a 8mm camera port is inserted with a 30 degrees endoscope and targeting the optimal surgical quadrant is achieved (Figure 1B). The procedure is performed using a monopolar scissors, a fenestrated bipolar and a megasuturecut needle driver with only 3 robotic arms docked having the fourth arm excluded.

### Technical considerations

**Figure 2:**
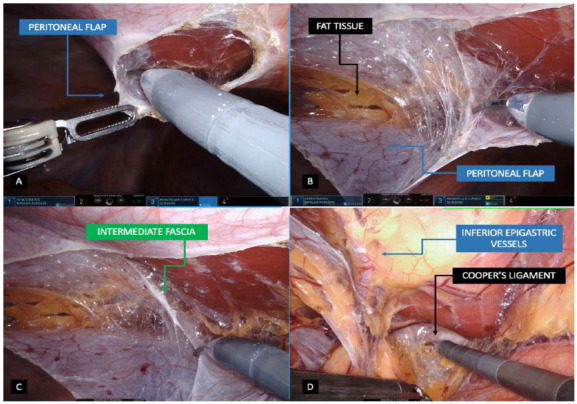
2A: Opening of the peritoneal flap in a medial to lateral approach / 2B: Peritoneal flap dissection leaving the fat tissue close to the abdominal wall / 2C: Intermediate fascia identified, defining the parietal and visceral compartment in medial and lateral areas respectively / 2D: Medial dissection reaching the Coopers ligament and assuring a wide space for mesh overlap.

**Figure 3:**
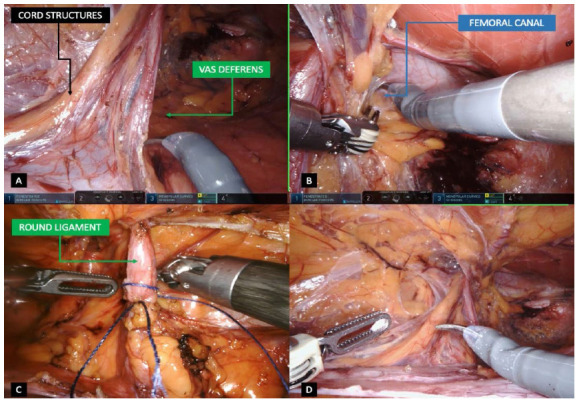
3A - Cords elements parietalized and vas deferens dissection. 3B - Dissection into the femoral orifice to rule out femoral defect. 3C - Round ligament ligation and transection in women’s repair. 3D - Visualization of the critical view of the myopectineal orifice.

**Figure 4:**
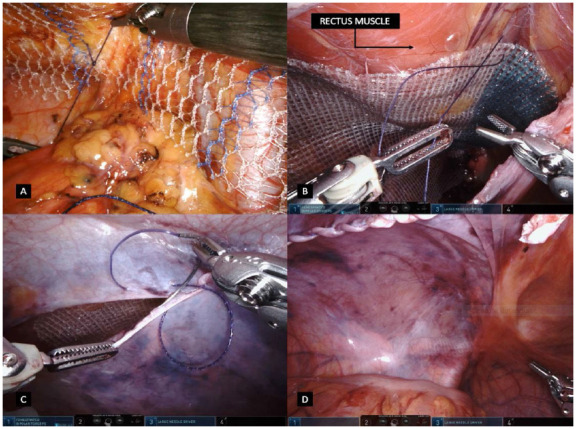
4A - Figure 4. 4A - Mesh fixation in the medial are, adminiculum lineae albae. 4B - Mesh suturing in the superior and medial aspect of the rectus muscle. 4C - Closure of the peritoneal flap. 4D - Direct view deflation of the peritoneal flap and mesh accommodation.

Once ports are correctly positioned, a more accurate inspection of the hernia defect is done. If an intraperitoneal hernia content is still present inside the inguinofemoral defect, the reduction is completely performed whenever possible. Peritoneal flap is created approximately 4cm from the deep inguinal ring in a medial to lateral approach, from the medial umbilical ligament to the anterior superior iliac spine direction in a longitudinal direction (Figure 2A). Applied to bilateral defects, a single peritoneal flap is created opening the whole peritoneal tissue connecting both sides by sectioning the medial and median umbilical ligaments is also possible, assuring a wide view of the posterior inguinofemoral anatomy. By pulling the peritoneal flap down, the carbon dioxide inside the peritoneal cavity enters the extraperitoneal space allowing an easier dissection of the surgical field. The preperitoneal space is dissected assuring that adiposity tissue is kept in contact with the abdominal wall and not close to the peritoneum fascia (Figure 2B). Medial and lateral compartments are connected after partial transection of the intermediate fascia; ensuring that medial and lateral compartments are dissected in the parietal and visceral planes respectively (Figure 2C). Medial dissection is performed at least 2cm medial to the rectus sheath confluence and 2cm below the pubis for sufficient space, having a good Cooper’s ligament (CL) exposure and space to accommodate an adequately sized mesh (Figure 2D). Lateral dissection is also achieved 2cm lateral to the ASIS and exposing the complete myopectineal orifice. Direct and indirect contents are dissected and reduced, having the cord elements parietalized and the vas deferens immersing into the pelvis, crossing the external iliac vein (Figure 3A). Dissection is achieved between CL and the iliac vein to identify the femoral orifice and a possible femoral hernia (Figure 3B). Cord lipomas are identified and reduced if present. Regarding direct defects, the medial aspect of fascia transversalis is not sutured or plicated due to potential risk of nerve injuries. Whenever confronted with women patients, the transection of round ligament is normally performed far from the deep inguinal ring to assure a correct and flat mesh placement, which can be difficult due to its adherence to the peritoneum (Figure 3C). Mesh placement is achieved by pulling out the right arm instrument and introducing the prosthesis inside the abdominal cavity. A hermetic and not folded mesh is positioned covering the entire myopectineal orifice with adequate overlap after hemostasis revision. The type of mesh placed may vary, however its dimensions should be at least 15x12cm coverage. Larger meshes are recommended especially in larger direct defects or in enlarged deep inguinal rings in inguinoscrotal hernias. Intraoperative dissection is achieved assuring the correct visualization of the critical view of the myopectineal orifice (Figure 3D). Mesh fixation is achieved using a 3-0 absorbable suture at the medial edge close to the pubis and CL, in the adminiculum lineae albae and superiorly close to the rectal muscle fibers; and laterally above the iliopubic tract to avoid nerve injuries (Figure 4A, B). Mesh fixations are performed even if a self-fixation mesh is used, especially in the adminiculum lineae albae due to its relative minor self-adherence into bone structures. Peritoneal flap is finally closed using a sutured using a 3-0 barbed suture having a decrease of the pneumoperitoneum pressure to 8-10 mmHg also helpful when approximating the peritoneal edges (Figure 4C, D). Just before the last suture to close the peritoneum, air aspiration in the pre-peritoneal space is carried out in order to adjust the peritoneal flap together with the prosthesis hermetically. Ports are retracted under direct vision and the supraumbilical port is closed using 0 absorbable suture.

## RESULTS

This described technique has been used by a single surgical group whose casuistry presents one of the most expressive in Brazil. Between March 2016 to February 2020, 97 patients were submitted to a robotic TAPP inguinal hernia repair. Most patients were males, 88 cases (90.7%) with mean age of 36.4 years (range: 22-71) and mean body mass index (BMI) of 26.9 kg/m2 (range: 19.9-35.2kg/m²). Patient´s demographic and perioperative variables are in Table 1. Seventy-eight patients (80.4%) had preoperative unilateral defects with 19 (19.6%) presenting bilateral hernias. Patients with primary defects were 74 (76.2%) while patients presenting hernia recurrence after previous repair were 23 cases (23.8%). Intraoperatively, an incidental contralateral or femoral defect was diagnosed in 9 and 4 cases respectively. No incidental intraoperatively contralateral defect was repaired. Mean mesh coverage area was 197 cm^2^ (range: 180 - 216 cm^2^). Robotic surgery was performed in all cases and da Vinci Xi platform represented 39 (40.2%) of the procedures while 58 (59.8%) were performed in the Si technology. Mean console time was 58 minutes (range: 40-150 min). None of the patients were converted to laparoscopic or open technique. Patients were discharged within 24 hours of their stay in a majority of cases and there was no 30-days mortality rate. Postoperative outcomes are described in Table 2. By actively questioning, neither neuralgia or testicle pain related to surgery were mentioned during routine appointments. No surgical site infection, hematoma or ischemic orquitis occurred. One patient had seroma fluid diagnosed with conservative management and complete recovery. Also, one patient had urinary retention requiring placement of a urinary catheter to relieve the distress without further complications. There was no major complication or recurrence rate within the median follow-up period of 642 days (range: 105 - 1450 days). 

**Table 1:** Patients demographics and characteristics.

Patients demographics	n (%)
Number of patients, (%)	97 (100)
Gender male/female, (%)	88 (90.7) / 9 (9.3)
Age, mean (range)	36.4 (range: 22 - 71)
Body mass index, mean (kg/m^2^)	27.7
ASA score, n (%)	
- I	71 (73.1)
- II	26 (26.9)
- III	0 (0)
- IV or V	0 (0)
Hernia defects number, n (%)	
- Unilateral	78 (80.4)
- Bilateral	19 (19.6)
Hernia defects type, n (%)	
- Primary	74 (76.2)
- Recurrence	23 (23.8)
Console time (minutes), mean (range)	58 (range: 40-150 min)
Robotic platform, n (%)	
- Si	58 (59.8)
- Xi	39 (40.2)
Mean mesh coverage area (cm^2^), mean (range)	(range: 180-216 cm²)

Data are presented as the number of patients (%) or mean (standard deviation) unless otherwise stated. ASA American Society of Anesthesiologists.

**Table 2:** Patients outcomes.

Patients outcomes	n (%)
Clavien - Dindo classification	
- I	2 (2.06)
- II	0 (0)
- IIIa	0 (0)
- IIIb / IVa / IVb / V	0 (0)
Surgical site infection	0 (0)
Seroma	1 (1.03)
Hematoma	0 (0)
Prolonged Ileus	0 (0)
Bowel obstruction	0 (0)
Neuralgia	0 (0)
Ischemic orchitis	0 (0)
Cardiovascular complications	0 (0)
Pulmonary complications	0 (0)
Renal complications	0 (0)
Urinary retention	1 (1.03)
Length of hospital stay (days)	1
30-day readmission	0 (0)
Recurrence	0 (0)
Mean follow-up (days), mean (range)	(range: 105-1450 days)

## DISCUSSION

Inguinal hernias repairs are still one of the most common procedures worldwide in abdominal surgery. Laparoscopic repair of groin hernia is safe and effective presenting several clear advantages over open repair, including less pain, quicker recovery time and better cosmetic outcome^8^. Transabdominal preperitoneal (TAPP) and total extra-peritoneal (TEP) differ although both techniques are in widespread use. The TAPP approach confers a theoretical advantage favoring an easier identification of anatomy with the starting intraperitoneal laparoscopy and allows identification of intraperitoneal hernia content, type or presence of a contralateral side defect^9^. 

Despite the evident benefits of minimally invasive surgery, it has been reported that laparoscopic inguinal hernia repair is not widely practiced^10^. First, a proper familiarity with the posterior view regarding inguinofemoral anatomy demands extensive practice and a way out from surgeon’s comfort zone. Second, skilled minimally invasive surgeons can achieve gentle dissection and utilizing conventional laparoscopy however not easily reproducible and insufficient in more complex cases. 

The robotic technology offers an enhanced visualization, superior dexterity and precision allied to wristed instruments to perform minimally invasive operations with finesse^11^. Benefits in oncological procedures regarding visceral surgery, urology and colorectal fields have already been described^12^ ^,^ ^13^. Concerning ventral hernia repairs, the robotic platform has shown encouraging outcomes allowing even more complex abdominal wall reconstructions in a minimally invasive approach^14^ ^,^ ^15^. In the inguinal hernia scenario, its application, safety and feasibility in robotic TAPP inguinal hernia repair is reported in the literature and has had a recent rapid growth^16^. 

Currently, the issues confronting robotic assisted procedures are especially related to operative times and costs. Similar to what has been discussed three decades ago, the introduction of laparoscopic approaches in the 90s also presented a more timing consuming procedure time and higher costs. In the groin hernia scenario, criticism was made even in the anaesthesiology field due to the requirement of a general anaesthesia instead of a spinal anesthesia. Cholecystectomies commonly performed by open approaches evolved to minimally invasive procedures after huge efforts proving its benefits with shorter hospital stays, reduced morbidity, more rapid return to work, and lower mortality as well-being considered cost-effective lately^17^. Presently, the gold standard laparoscopic approach in cholecystectomies is unquestionable but its potential benefits were strongly confronted during initial practice. Historically, adoption of technology in the surgical field requires long adaptations and not often coalesce into coherent knowledge^18^. 

Currently, literature comparing laparoscopic with robotic inguinal hernia repairs is still scarce. Robotic approach is thought to be associated with longer operative time and room time when compared with the laparoscopic^19^. Robotic docking by untrained surgical groups as well as mastering surgeons’ familiarity to the robotic platform and its learning curve could corroborate it. Also, not only hernia related, one of the biggest concerns over performing robotic surgery is cost. As the demand for innovation and technology in healthcare assistance increases, so does its costs. During the initial MIS era, laparoscopic cameras, instruments, towers and operating room tables were also more expensive and required initial capital expenditures. What initially was tough to be not affordable and rich, further analysis reported to be cost-effective. Analogously, it’s possible that the current robotic-assisted surgery era could be facing the same questioning. Capital costs are amortized over time and over all patients treated by the platform, not only focused on abdominal wall procedures, but also by all the robotic surgical fields. Regarding robotic inguinal hernia repairs, mesh fixation is normally performed by sutures not making necessary the use of endoscopic tacks. and the peritoneal flap is closed in a running suture. Eliminating the tacker could represent a cost benefit besides possible association to lesser pain related to mesh fixation or peritoneal close^19^. Also, in procedures regarding recurrent hernia defects, with prior mesh in the anterior or posterior anatomy, our experience step up for the robotic platform allowing a more precise dissection between cord structures and mesh or previous adherences. This case series reported no neuralgia or chronic pain related to the procedure, reliable to what’s reported in literature^20^ ^,^ ^21^. Conceptually, there may be less risk of nerve injury and chronic pain by avoiding tacks and trauma to the abdominal wall musculature^22^. Grossi et al.^23^ even described identification of the nerves during minimally invasive approaches. Also, robotic surgery allows the trocar sites to have a fixed pivot point and results in less trocar site torque, possibly causing less trauma to the abdominal wall. 

Through this study, we found that the robotic approach is certainly feasible, reproducible and shows encouraging postoperative outcomes. As surgeons maintain and increase their armamentarium and robotic skills, more complex and challenging operations initially not though suitable to a minimally invasive surgery could be overcomed to robotic approach. The outcomes reported should step up to continued investigation of robotic surgery applied to abdominal wall techniques without excessive concern over its cost. As the adoption of robotic technology in general surgery continues to grow worldwide, surgeons experienced with minimally invasive surgery should familiarize with the robotic inguinal repair technique. Through the previous described guided surgical standard procedure, this article may elucidate and bring robotic surgeons trainees more reliability to robotic TAPP inguinal hernias repairs. 

Although this article presents some limitations due to its intrinsic retrospective analysis and limited to a single referred surgical group experience, its results reported are encouraging. Despite these limitations, this study shows that safe outcomes can be achieved by trained surgical groups familiarized to the robotic platform and understanding the posterior anatomy of the groin area. However, prospective and possibly multi-institutional studies are needed to evaluate robotic inguinal hernia repairs further.

## CONCLUSION

To the best of our knowledge, this is the first Brazilian case series to date of robotic TAPP inguinal hernia repair and shows encouraging outcomes with a safe and reproducible technique. The robotic TAPP technique in inguinal hernia repair could provide benefits in selected patients with reduced pain, lower recurrence rates and long-term quality of life. Future prospective studies and randomized controlled trials could elucidate its real benefits in inguinal hernia repairs.
